# ETE 3: Reconstruction, Analysis, and Visualization of Phylogenomic Data

**DOI:** 10.1093/molbev/msw046

**Published:** 2016-02-26

**Authors:** Jaime Huerta-Cepas, François Serra, Peer Bork

**Affiliations:** ^1^Structural and Computational Biology Unit, European Molecular Biology Laboratory, Heidelberg, Germany; ^2^Centro Nacional de Análisis Genómico (CNAG-CRG), Center for Genomic Regulation, Universitat Pompeu Fabra (UPF), 08028 Barcelona, Spain; ^3^Germany Molecular Medicine Partnership Unit (MMPU), University Hospital Heidelberg and European Molecular Biology Laboratory, 69117 Heidelberg, Germany; ^4^Max Delbrück Centre for Molecular Medicine, 13125 Berlin, Germany

**Keywords:** phylogenomics, tree visualization, tree comparison, NCBI taxonomy, hypothesis testing, phylogenetics.

## Abstract

The Environment for Tree Exploration (ETE) is a computational framework that simplifies the reconstruction, analysis, and visualization of phylogenetic trees and multiple sequence alignments. Here, we present ETE v3, featuring numerous improvements in the underlying library of methods, and providing a novel set of standalone tools to perform common tasks in comparative genomics and phylogenetics. The new features include (i) building gene-based and supermatrix-based phylogenies using a single command, (ii) testing and visualizing evolutionary models, (iii) calculating distances between trees of different size or including duplications, and (iv) providing seamless integration with the NCBI taxonomy database. ETE is freely available at http://etetoolkit.org

The Environment for Tree Exploration (ETE) is a toolkit developed to facilitate the computation, analysis and visualization of phylogenetic data. ETE provides a comprehensive Python programming library (API) that allows researchers to automate common tasks in comparative genomics. Since its first release (Huerta-Cepas et al. 2010), ETE has been widely used as a computational framework to perform numerous phylogenomic analyses, including characterizing newly sequenced genomes (Richards et al. 2010; Wang et al. 2014), extracting information from large sets of phylogenetic trees (Derelle and Lang 2012; Chiapello et al. 2015; Marcet-Houben and Gabaldón 2015) and developing third party tools and databases (Zhang et al. 2013; Huerta-Cepas et al. 2014; Szitenberg et al. 2015). Here, we describe the latest version of the software (ETE v3), featuring a significantly improved API library and a novel collection of standalone tools. While the API continues to offer full programmatic control on data analysis and visualization, the new standalone tools facilitate the use of common phylogenetic methods at the genomic scale. We here describe the most notable additions.

## Tree Building

**Fig. 1:**
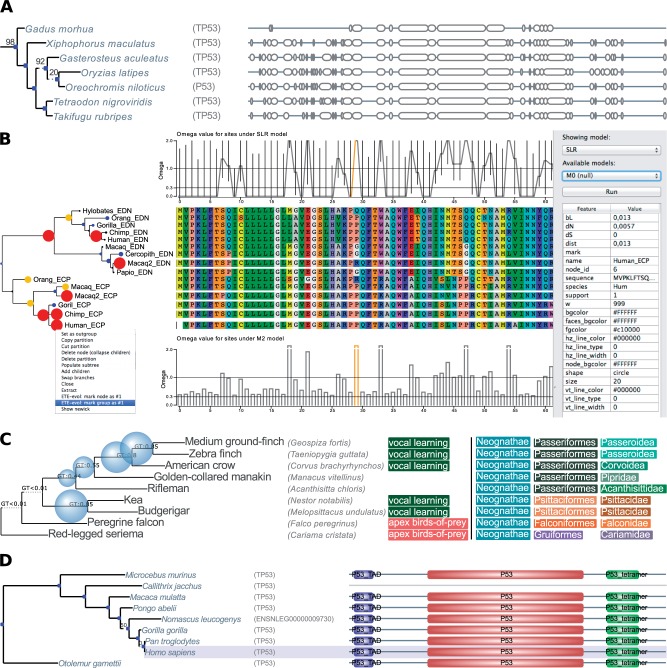
Several phylogenetic tree images generated using the ETE toolkit. (*A*) Gene tree reconstructed using *ete-build.* The figure shows the relationships between several P53 genes together with their aligned sequences visualized in condensed format. (*B*) Tree image generated by *ete-evol* for three models fitted to a classical example (Bielawski and Yang 2004). (i) The line chart on top of the alignment indicates the omega estimates for sites as calculated by the SLR software. (ii) The bar chart at the bottom part shows the *dn/ds* ratio for each site under the M2 site-model from CodeML. Line colors in both charts indicate the significance of assigning a site to a given class of positive selection (i.e., red for *P*-value <0.01 and orange for *P*-value <0.05). (iii) The color and size of tree nodes represent the *dn/ds* ratio estimated for tree branches using the free-ratio model from CodeML. Blue small circles indicate a ratio between 0.2 and 1, medium yellow nodes indicate a ratio >1, and big red nodes for infinite values. Note that the right side panel allows users to select the models to be displayed, and even starting new runs using predefined models. (*C*) Portion of a recently published bird species tree (Jarvis et al. 2014) annotated with gene–tree support values (blue spheres), custom node labeling (first aligned column) and taxonomic information (next aligned columns). (*D*) Example of a phylogenetic tree visualized with a sequence alignment and domain composition as used in the eggNOG database (Huerta-Cepas et al. 2016).

The *ete-build* tool provides a unified interface to wrap the execution of reproducible phylogenetic workflows, comprising the reconstruction of gene–trees and supermatrix-based species trees. To do so, ETE relies on a versioned collection of external tools that are transparently installed and executed upon request. A single command is used to configure and launch complex phylogenetic pipelines, covering sequence alignment, trimming, substitution-model testing, tree inference, and image rendering (fig. 1*A*). In addition, the supermatrix-based reconstruction mode permits to build and concatenate multiple sequence alignments with ease, simplifying the inference of species trees based on multiple genes. Advanced options allow to automatically switch from amino-acid to nucleotide alignments based on sequence identity, resuming the execution of workflows, or even testing multiple strategies in parallel. As an example, a single command line can be used to test several alignment methodologies or phylogenetic inference programs simultaneously, making the tool particularly suitable to run phylogenomic pipelines. Notably, ETE-build was recently used to compute over one million phylogenetic trees for the EggNOG v4.5 database (Huerta-Cepas et al. 2016).

## Testing Evolutionary Hypotheses

Measuring selective pressures on molecular sequences is a common task in evolutionary biology. Softwares such as CodeML (Yang 2007) or SLR (Massingham and Goldman 2005) provide the statistical and computational framework to perform these analyses. However, the use of such tools at the genomic scale requires substantial work on data preparation, on experimental design, and on results interpretation. To aid in these tasks, the *ete-evol* tool automates CodeML/SLR-based analyses by using pre-configured evolutionary models and directly producing a graphical representation of the results. These pre-configured models include site (Yang et al. 2000; Massingham and Goldman 2005), branch (Yang and Nielsen 2002), branch-site (Zhang et al. 2005), and clade (Yang and Nielsen 2002; Bielawski and Yang 2004) models. For instance, *ete-evol* can test, in parallel, and with a single call, the differential selective pressures along each branch in a given phylogeny. Importantly, fitted models are compared using a built-in likelihood ratio test. Evolutionary measures from the best-fitting models are then plotted (or interactively visualized) by mapping the predicted selective pressures acting on sites and branches into the tested topology, as well as on the multiple sequence alignment (fig. 1*B*). For convenience, raw output files produced by CodeML and SLR can also be visualized using *ete-evol*.

## Comparing Trees

ETE v3 provides three measures to compute distances between trees, namely the Robinson–Foulds distance (Robinson and Foulds 1981), a branch congruence measure (%) and the TreeKO Speciation distance (Marcet-Houben and Gabaldón 2011). In contrast to existing software (Felsenstein 2005; Soria-Carrasco et al. 2007), *ete-compare* calculates all three distances at the same time; it accepts trees varying in size and containing duplication events; it allows filtering branches with low support; and it is optimized for comparing large datasets. In addition, *ete-compare* can provide a detailed list of the differences and coincidences among the compared trees for further analysis. Conveniently, the TreeKO method for splitting gene trees into duplication-free subtrees has been optimized and integrated into ETE’s API library, thereby enabling its use for other tests. For instance, ETE allows summarizing the phylogenetic signal (i.e., gene tree support) from an heterogenous sample of gene trees using a species tree topology as reference (fig. 1*C*).

## Taxonomy Databases

Efficient queries to the NCBI-taxonomy database (Benson et al. 2014) are now available through the *ete-ncbiquery* tool or the relevant methods in the API. Extracting pruned subtrees, converting NCBI *taxids* into their corresponding scientific names, obtaining full lineage tracks, or annotating user-trees with taxonomic data, are common tasks that can be easily performed with the *ete-ncbiquery* tool. Importantly, all queries are carried out locally, avoiding unnecessary lags and permitting the integration of the tool into genomic and metagenomic pipelines.

Finally, other ETE-tools and methods are available that aid in routine tasks such as format conversion, topology manipulation, and custom visualization of trees linked to multiple sequence alignments (fig. 1*D*).

## Conclusions

Although several software packages are available for the standalone exploration of trees (Letunic and Bork 2007; Huson and Scornavacca 2012; Asnicar et al. 2015) and the programmatic manipulation of data (Paradis et al. 2004; Knight et al. 2007; Sukumaran and Holder 2010; Vos et al. 2011; Talevich et al. 2012), ETE offers a unified framework to compute and analyze genome-wide collections of evolutionary data while providing unique visualization capabilities. Moreover, with the recent addition of the command line tools, ETE has significantly broadened its scope, simplifying many common tasks in phylogenomics for both expert and casual users.
